# Angiographic Characteristics of the Vein of Marshall in Patients with and without Atrial Fibrillation

**DOI:** 10.3390/jcm11185384

**Published:** 2022-09-14

**Authors:** Lei Ding, Hongda Zhang, Fengyuan Yu, Lijie Mi, Wei Hua, Shu Zhang, Yan Yao, Min Tang

**Affiliations:** Department of Cardiology, State Key Laboratory of Cardiovascular Disease, Cardiovascular Institute, Fuwai Hospital, National Center for Cardiovascular Diseases, Chinese Academy of Medical Sciences, and Peking Union Medical College, Beijing 100037, China

**Keywords:** atrial fibrillation (AF), vein of Marshall (VOM), ethanol infusion, coronary sinus (CS), coronary sinus ostium (CSo)

## Abstract

Background: Ethanol infusion into the vein of Marshall (Et-VOM) is a novel therapeutic treatment for atrial fibrillation (AF). However, few studies have focused on the difference between AF and non-AF patients (presented other types of arrhythmias) regarding the characteristics of the vein of Marshall (VOM). Objective: This study sought to investigate the incidence, morphology, and angiographic characteristics of the VOM. Methods: Coronary sinus (CS) angiography was performed in all patients. The baseline, angiographic characteristics and measurements of VOM dimensions were compared between the AF and non-AF group. Results: CS angiography was performed in 290 patients. The VOM detection rate was higher in the AF group than in the non-AF group (91.8% vs. 84.1%, *p* = 0.044). In the right anterior oblique (RAO) projection, AF patients had significant larger VOM ostium, CS ostium, and CS diameter at VOM ostium than non-AF patients (1.9 ± 0.9 vs. 1.7 ± 0.7 mm, *p* = 0.015; 12.8 ± 4.1 vs. 11.4 ± 3.7 mm, *p* = 0.016; 9.1 ± 3.1 vs. 8.2 ± 2.9 mm, *p* = 0.028, respectively). There was a slight linear correlation between the VOM ostium and the CS ostium diameter as well as left atrial volume (LAV). Conclusion: AF patients seem to have a higher incidence of the VOM, larger VOM ostium, CS ostium, and CS lumen in RAO view. Meanwhile, the VOM ostium may correlate with the CS ostium and LAV.

## 1. Introduction

Radiofrequency catheter ablation (RFCA) has been a well-established treatment for atrial fibrillation (AF) [1,2,3]. Pulmonary veins (PVs) are the most common ectopic triggers for initiating AF, and the treatment strategy mainly consists of PV isolation (PVI). However, only PVI are inadequate when dealing with persistent AF. Many patients suffer recurrent AF and atrial tachycardia which need a redo procedure.

The vein of Marshall (VOM) is an embryological remnant of the left superior vena cava and has been recognized as an important factor for AF initiation and maintenance [4,5]. In addition, the VOM is an epicardial substrate for maintaining perimitral atrial tachycardia [6]. Previous studies have reported the efficacy and safety of ethanol infusion in the VOM [7,8,9,10]. Chemical ablation by ethanol infusion could eliminate non-PV AF triggers and facilitate a bidirectional block of the mitral isthmus [11,12]. However, there are no detailed reports in a large cohort of patients with and without AF on the characteristics of the VOM. Meanwhile, the influence of VOM morphology on Et-VOM is also absent. 

This study aimed to investigate the incidence, morphology, and angiographic characteristics of the VOM in patients with and without AF by angiography.

## 2. Materials and Methods

### 2.1. Population

This was a prospective, cohort study enrolling all patients with tachyarrhythmias who underwent RFCA in Fuwai Hospital between November 2021 and April 2022. No specific exclusion criteria were used. Clinical data, echocardiographic, and imaging data were documented in the study. All patients provided informed consent prior to the procedure. The study protocols were approved by the Ethics Committee of Fuwai Hospital, Chinese Academy of Medical Sciences and were in accordance with the Declaration of Helsinki (No. 2022-1810). Antiarrhythmic drugs were discontinued for at least five half-life periods prior to the procedure.

### 2.2. Electrophysiology Study and Catheter Ablation

All catheters were inserted percutaneously by the Seldinger technique and were advanced into position under fluoroscopic guidance. A 6F decapolar steerable catheter (Triguy, APT Medical, CHN) was inserted into the coronary sinus (CS) through the left femoral vein. Two quadripolar catheters (6F, Triguy, APT Medical, CHN) were advanced into the His bundle position and the right ventricle in patients with supraventricular tachycardia (SVT). The surface electrocardiogram and intracardiac electrograms were continuously monitored and stored in a recording system (C. R. Bard, Inc., Lowell, MA, USA). Left atrial access was attempted by transseptal puncture when needed, and heparin was given. The mapping catheter was advanced via a SL1 long sheath. Each patient underwent the standard electrophysiology study, including programmed stimulation and diagnostic pacing maneuvers [13]. Isoproterenol was administered for patients who could not induce tachycardia by standard procedures. Radiofrequency (RF) energy was delivered from a generator (Stockert, Freiburg, Germany). The RF power setting followed the standards of the different procedures, ranging from 25 to 45 W.

AF ablation mainly consisted of antral PVI and was performed under sedation using midazolam and fentanyl. For persistent AF patients and paroxysmal AF patients who were in AF rhythm before ablation, VOM angiography and ethanol infusion were performed. After ethanol infusion, mitral isthmus was ablated to achieve a bidirectional conduction block. The end points of the procedure were as follows: (1) atrioventricular nodal reentrant tachycardia (AVNRT)—either a slow pathway blocked or a single atrial echo beat at baseline and during isoproterenol infusion without inducing tachycardia; (2) atrioventricular reentrant tachycardia (AVRT)—appearance of decremental antegrade and retrograde atrioventricular conduction and could not induce tachycardia; (3) atrial flutter (AFL)—a bidirectional conduction block confirmed by differential pacing; (4) AF—absence of ostial PV potentials on a circumferential mapping catheter and no atrial capture from multiple pacing sites within the PV ostium. End points had to be fulfilled after a waiting period of 20 min after the last ablation. Additionally, for patients diagnosed with paroxysmal AF, 40 mg of adenosine triphosphate (ATP) and isoproterenol were given to verify the block of PVs and induce AF. 

### 2.3. VOM Angiography and Ethanol Infusion

**Figure 1:**
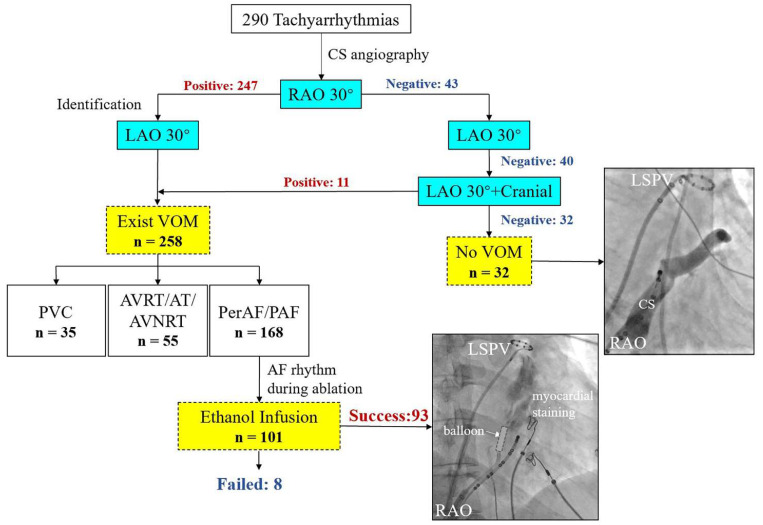
Flowchart of CS angiography, VOM angiography, and ethanol infusion procedure. AF = atrial fibrillation; AT = atrial tachycardia; AVNRT = atrioventricular nodal reentrant tachycardia; AVRT = atrioventricular reentrant tachycardia; CS = coronary sinus; LAO = left anterior oblique; LSPV = left superior pulmonary vein; RAO = right anterior oblique; PAF = paroxysmal atrial fibrillation; PerAF = persistent atrial fibrillation; PVC = premature ventricular contraction; VOM = vein of Marshall.

After pulmonary vein isolation, the CS was canulated with a SL1 long sheath (8.5F, St. Jude Medical, Inc., St Paul, MN, USA) or a steerable long sheath (8.5-F, Agilis NxT; Abbott) inserted from the right femoral vein. A guiding catheter (6-F Judkins Right [JR] 4; Medtronic, Minneapolis, MN, USA) was proximally positioned inside the CS lumen, and contrast was injected to perform CS angiography. Details of stepwise angiography to identify the VOM were shown in Figure 1. First, the CS angiography in the right anterior oblique (RAO) 30° was used. The proximal portion of the valve of Vieussens was targeted and the JR 4 guiding catheter was rotated posteriorly and superiorly to find the ostium of the VOM [14]. In addition, at each location, a small amount of contrast was injected through the guiding catheter to confirm the VOM. Second, if the operator found the suspected VOM, the left anterior oblique (LAO) 30° was performed to identify. However, if there was no suspected VOM, the LAO 30° or LAO 30° with cranial 30° was used to find the VOM. For patients in non-AF group, the VOM angiography was conducted during the waiting period of 20 min.

After identifying the VOM, an angioplasty guidewire (Sion Blue 0.014 inch, Asahi) was advanced into the lumen of the VOM with an over-the-wire balloon preload at the distal portion. An appropriately sized balloon (1.5–2 mm diameter and 8–15 mm length; Boston Scientific, USA) was used depending on the size of the VOM. Subsequently, the balloon was inflated, starting at 1–2 atm, until the operator felt some resistance on the inflator with a maximum of 8–10 atm, and the guidewire was removed. After confirming balloon occlusion, 2 mL of ethanol (95%) was slowly administered over one minute, and the selective angiogram of the VOM was repeated to confirm the myocardial lesion created by ethanol [15]. Following every injection, the balloon was deflated and retracted 1 cm towards the proximal VOM; in the same way, another injection was performed with 2 mL of ethanol. The balloon was retracted until it reached the VOM ostium and a total of 10–12 mL of ethanol was used as a maximum dose.

### 2.4. Measurements and Definitions 

**Figure 2:**
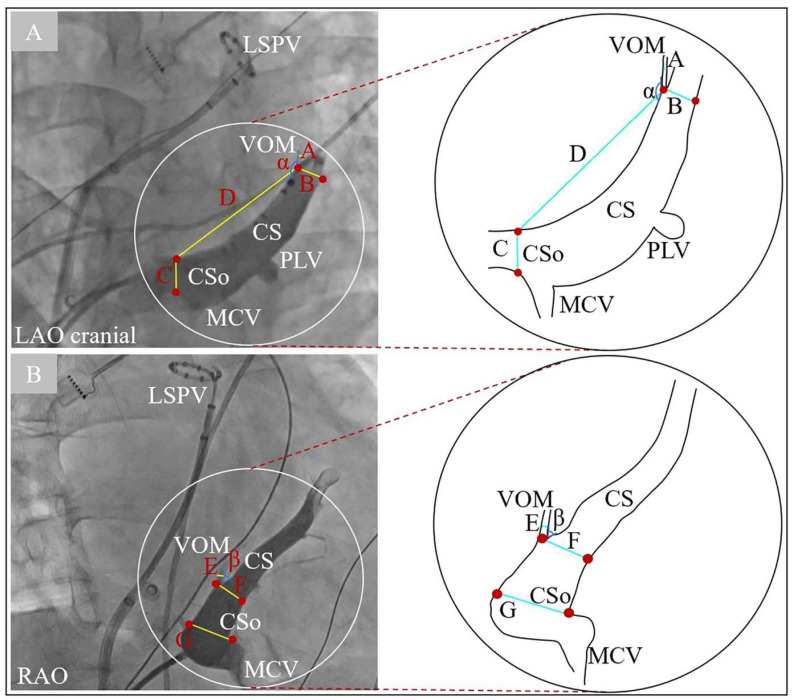
Measurements of the VOM in the LAO/LAO cranial (**A**) and RAO (**B**) views by CS angiography. Segment A and E = diameter of VOM; segment B and F = diameter of the CS at VOM level; segment C and G = diameter of CSo; segment D = distance between VOM ostium and CSo; angle α = angle between VOM and proximal CS; angle β = angle between VOM and distal CS. CS = coronary sinus; CSo = coronary sinus ostium; LAO = left anterior oblique; LSPV = left superior pulmonary vein; MCV = middle cardiac vein; PLV = posterior lateral vein; RAO = right anterior oblique; VOM = vein of Marshall.

**Figure 3:**
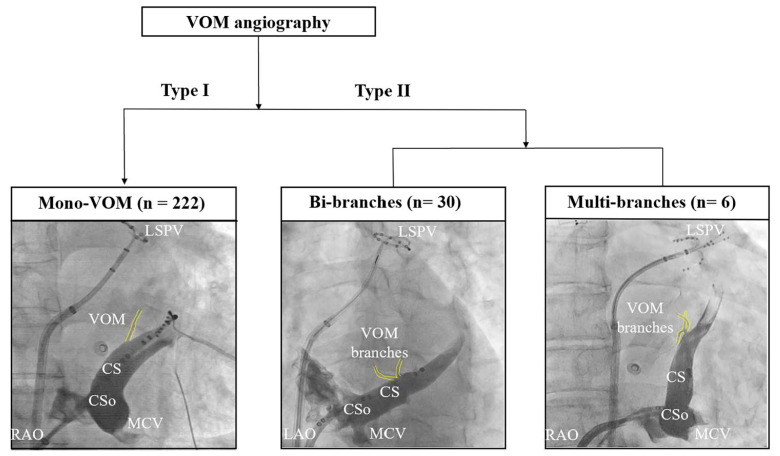
Morphologic classification of the VOM. CS = coronary sinus; CSo = coronary sinus ostium; LAO = left anterior oblique; LSPV = left superior pulmonary vein; MCV = middle cardiac vein; RAO = right anterior oblique; VOM = vein of Marshall.

Measurements of the VOM were performed in the RAO 30° and LAO 30° or LAO 30° with cranial 30° (Figure 2). The diameters of the CS ostium (CSo), the VOM ostium, and CS lumen at the VOM ostium were measured in all views. In addition, in the LAO 30° or LAO 30° with cranial 30° (Figure 2A), the distance between the VOM ostium and the CSo, and the angle between the VOM lumen and proximal CS lumen, were measured. Subsequently, in the RAO 30° (Figure 2B), operators measured the angle between the VOM lumen and distal CS lumen. According to the number of branches at the VOM ostium, we classified all VOM into two categories (Figure 3). Type I VOM was defined as only one branch and Type II VOM was defined as more than one branch within 1 mm around the VOM ostium.

### 2.5. Statistical Analysis

Statistical analysis and graphing were performed by SPSS IBM 22 (IBM Co., Armonk, NY, USA) and GraphPad Prism 8.0 (GraphPad Software Inc., La Jolla, CA, USA). Continuous data are presented as means ± standard deviation (SD) or the median and interquartile range, depending on the normality of the distribution. Categorical variables are described as frequency counts and percentages. For continuous data, either the student *t*-test or Mann-Whiney *U*-test was carried out for statistical comparisons. For comparisons of categorical data, the chi-squared test was performed. A *p* value less than 0.05 indicated statistical significance.

## 3. Results

CS angiography was performed in 290 consecutive patients (age 55.2 ± 13.2 years; 114 females) scheduled for RFCA between November 2021 and April 2022. Of these, 183 (63.1%) patients had AF, 36 (12.4%) had AFL, 29 (10.0%) had AVNRT, 20 (6.9%) had AVRT, 20 (6.9%) had AT, and 47 (16.2%) had ventricular arrhythmias. The AF group consisted of 183 patients, whereas the non-AF group consisted of 107 patients. In the non-AF group, there were no patients that presented previous AF episodes. The baseline characteristics of all patients are summarized in Table 1. Of all the AF patients, there were 85 paroxysmal AF (PAF), 98 persistent AF (PerAF), and 14 patients also had AFL. Patients with AF were significantly older, had a higher rate of hypertension and heart failure, and a higher left atrial volume (LAV) than non-AF patients. Similarly, left ventricular ejection fraction (LVEF’s) were significantly lower in the AF group compared with the non-AF group. Meanwhile, the CHA_2_DS_2_-VASc score and the HAS-BLED score were 2.0 ± 1.5 and 0.6 ± 0.7, respectively, in AF patients. Other clinical characteristics are detailed in Table 1.

There were 101 patients that underwent ethanol infusion into the VOM (Et-VOM); a total of 8 patients failed Et-VOM and the success rate of Et-VOM was 92.1% (Figure 1). Measurements of angiographic images are shown in Table 2. The VOM detection rate was significantly higher in the AF group than in the non-AF group (91.8% vs. 84.1%, *p* = 0.044). Of all undetectable cases (n = 32), four patients were diagnosed as persistent left superior vena cava (PLSVA) with no statistically significant difference between the two groups (1.1% vs. 1.9%, *p* = 0.584). There was no significant difference between patients with and without AF in morphologic classifications of VOM (*p* = 0.477). In measurements in the RAO view, AF patients had a significantly larger VOM ostium diameter, CSo diameter, and CS diameter at the VOM ostium than non-AF patients (1.9 ± 0.9 vs. 1.7 ± 0.7 mm, *p* = 0.015; 12.8 ± 4.1 vs. 11.4 ± 3.7 mm, *p* = 0.016; 9.1 ± 3.1 vs. 8.2 ± 2.9 mm, *p* = 0.028, respectively), while the VOM-CS angle was similar in both groups. For measurements in the LAO view, AF patients had a smaller VOM-CS angle and larger CSo diameter as well as CS diameter at the VOM level (144.2 ± 36.8° vs. 156.0 ± 12.6°, *p* = 0.036; 11.7 ± 5.8 vs. 10.1 ± 5.4 mm, *p* = 0.022; 7.2 ± 2.9 vs. 6.5 ± 2.0 mm, *p* = 0.047). Other details are shown in Table 2.

The non-AF patients (n = 76) and AF patients (n = 76) were then matched in a ratio of 1:1 based on age and gender. As a result, there were no significant differences between the two groups except LAV (68.7 ± 25.3 mL vs. 52.2 ± 19.5 mL, *p* < 0.001, Supplementary Table S2). Patients in the AF group still have a significantly higher incidence of the VOM, larger VOM ostium diameter, and CSo diameter in the RAO view (Supplementary Table S3).

**Figure 4:**
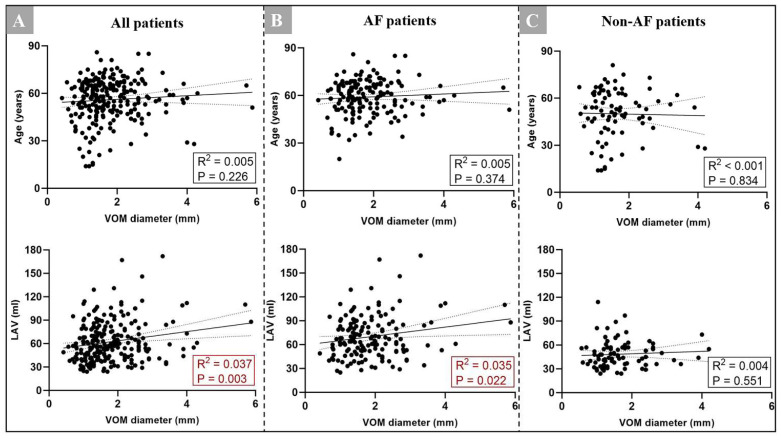
(**A**) Correlation between the VOM ostium diameter and age as well as LAV in all patients (n = 290); (**B**) correlation between the VOM ostium diameter and age as well as LAV in AF patients (n = 183); (**C**) correlation between the VOM diameter and age as well as LAV in non-AF patients (n = 107). AF = atrial fibrillation; CS = coronary sinus; CSo = coronary sinus ostium; LAV = left atrial volume; VOM = vein of Marshall.

**Figure 5:**
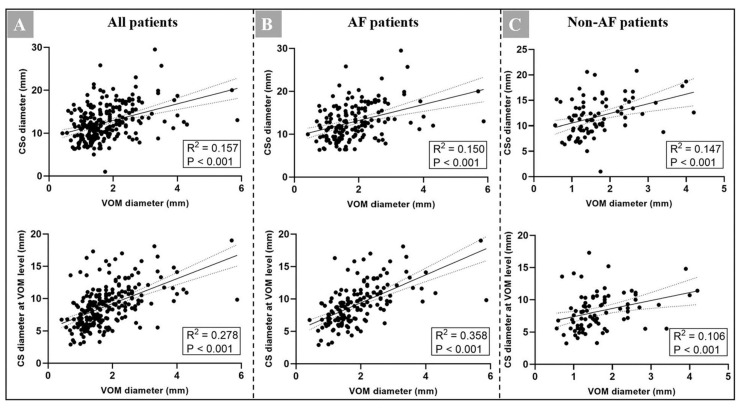
(**A**) Correlation between the VOM ostium diameter and the CSo diameter as well as CS diameter at the VOM level in the RAO plane in all patients (n = 290); (**B**) correlation between the VOM ostium diameter and the CSo diameter as well as CS diameter at the VOM level in the RAO plane in AF patients (n = 183); (**C**) correlation between the VOM ostium diameter and the CSo diameter as well as CS diameter at the VOM level in the RAO plane in non-AF patients (n = 107). AF = atrial fibrillation; CS = coronary sinus; CSo = coronary sinus ostium; RAO = right anterior oblique; VOM = vein of Marshall.

We initially investigated the linear correlation between the VOM ostium and age as AF patients were significantly older. We did not find a linear correlation between the VOM ostium and age in the AF group, non-AF group, or the total patient group (Figure 4). Meanwhile, we found that there were no significant differences of detection rate of the VOM, diameter of the VOM, VOM-CS angle, and VOM-to-CSo distance in all views between different genders (Supplementary Table S1). We also found that male patients have a significantly larger CSo diameter in RAO and LAO cranial views (11.6 ± 4.0 vs. 12.7 ± 4.0 mm, *p* = 0.025; 12.0 ± 4.2 vs. 13.4 ± 5.4 mm, *p* = 0.041). In addition, patients with a higher LAV may have a larger VOM in the AF group, while there was no obvious linear correlation in the non-AF group (Figure 4). We also found that patients with a larger CSo or CS diameter at the VOM ostium had a larger VOM ostium in both groups and had a slight linear correlation (*p* < 0.001, Figure 5).

## 4. Discussion

In summary, the present study illustrated the difference between AF and non-AF patients regarding the VOM dimensions. The main findings of this study are as follows: (1) AF patients were associated with a significantly higher incidence of the VOM, larger VOM ostium, CSo, and CS lumen at the VOM ostium; (2) there was a slight linear correlation between the diameter of the VOM ostium and CSo as well as the diameter of CS at the VOM level; (3) patients with a larger LAV tend to have a larger VOM ostium. 

### 4.1. Characteristics of the VOM and Difference between Patients with and without AF

According to previous reports, the incidence of the VOM varied from 21% to 98% in anatomical studies [16,17]. The presence of the VOM ranged from 63% in a study of optimized VOM-CT protocol to 73% [18] in a study of 100 patients undergoing CS angiography [19]. By contrast, in the present study, the overall incidence of the VOM is 89% under the workflow of angiography (Figure 1). As Et-VOM has been proved to benefit outcomes of AF and will be widely used [7,9], the angiography of workflow should be undergone before Et-VOM to identify the VOM, especially for centers that are in the early stage of operating Et-VOM. In addition, we found that patients with AF have a significantly larger VOM ostium, CSo, and CS lumen at the VOM ostium. The CSo diameter is comparable with previous studies [20,21,22]. Ortale et al. [22] reported that the CSo ranged from 4.0 to 16.0 mm in 37 adult human heart specimens. Another study [21] involved measurements of CSo by angiography and showed that the median diameter of CSo was 10–11 mm in non-AF patients. However, few studies compare the VOM between patients with and without AF. Our study demonstrated that the VOM ostium and the CSo are larger in AF patients and there may be a correlation between them. Results of the present study suggest that anatomic factors may contribute to the genesis of AF. Larger VOM may produce more atrial connections into the left atrium, thus, giving rise to AF. Although the pathogeneses of AF are complicated, other large scale, prospective cohort studies need to be conducted to prove these hypotheses.

### 4.2. The Morphology Characteristics of the VOM

Some studies have investigated the morphology of the VOM. Valderrábano et al. [23] pointed that the VOM was varied in branching patterns and 88.6% of patients present venous plexus at the VOM ostium or variable branches into small venules. Kamakura et al. [8] further classified the VOM into three morphologies according to the numbers of branches in the trunk. They found multiple branches in 92%, a large trunk in 5.0%, and no branches in 3.0%. In the present study, we classified the VOM as two categories according to the numbers of branches within 1 mm of the VOM ostium. Type I was over 70% (222/76.6%), while Type II only counts for 23.4%. However, there was no difference between AF and non-AF patients. 

### 4.3. Correlation between the VOM and LAV

Many studies had reported that the LAV contributes to the pathogenesis of AF [24,25,26,27,28]. An echocardiographic measurement of LAV was shown to be a predictor of new-onset AF in 574 subjects, and prospectively followed for a mean of 1.9 years [24]. Tan et al. [25] also pointed that the LAV index is associated with new-onset AF in embolic strokes of an undetermined source in patients. However, few studies have investigated the correlation between the LAV and left atrial veins. In our study, we found that AF patients tend to have a larger LAV, and there was a weak linear correlation between the LAV and the size of the VOM ostium. As Njoku et al. [26] concluded, a larger LAV may present structural remodeling, atrial hypertrophy, and stretch peri-LA tissues. In this way, an increased LAV may result in a larger VOM ostium. This morphologic variation may be the anatomic substrate for AF pathogenesis, but still needs to be investigated in other large-scaled, prospective cohort studies. 

### 4.4. Limitations

There are some limitations of this study. First, it is possible that the left atrial appendage (LAA) vein was mistaken as the VOM because it also drains into the CS. However, the LAA vein extends toward the anterior and we confirmed the course of the vein both at RAO and LAO views. Second, the incidence of the VOM remains unclear in the anatomical study; for patients who could not find the VOM, we changed at least two different sites and fluorography angles for angiography. Third, this study consisted of a small sample size, whereas a prospective cohort study with a larger population is necessary to evaluate the correlation between the VOM and LAV. Fourth, the two-dimensional (2D) imaging of angiography may could not assess a 3D structure, including the VOM, adequately; we used as many angiography views as possible to illustrate the characteristics of the VOM. Other techniques, such as 3D quantitative angiography, may mitigate against this limitation. 

## 5. Conclusions

AF patients appear to have a higher incidence of the VOM, larger VOM ostium, CSo, and CS lumen in the RAO view compared to non-AF patients. Meanwhile, the VOM ostium may correlate with the CS ostium and LAV.

## Figures and Tables

**Table 1:** Clinical characteristics of all patients.

	AF (n = 183)	Non-AF (n = 107)	*p* Value
Age onset, y	53.9 ± 11.6	45.4 ± 16.5	<0.001 *
Age at admission, y	58.5 ± 11.0	50.5 ± 15.1	<0.001 *
Female, n (%)	57 (31.1)	57 (53.3)	0.001 *
BMI, kg/m^2^	25.8 ± 3.6	25.0 ± 3.5	0.059
Comorbidities, n (%)			
Hypertension	95 (51.9)	35 (32.7)	0.002 *
Diabetes mellitus	36 (19.7)	18 (16.8)	0.547
Coronary heart disease	31 (16.9)	13 (12.1)	0.273
Heart failure	25 (13.7)	4 (3.7)	0.007 *
Stroke	24 (13.1)	7 (6.5)	0.080
Vascular disease	19 (10.4)	5 (4.7)	0.089
CHA_2_DS_2_-VASc score	2.0 ± 1.5	-	-
HAS-BLED score	0.6 ± 0.7	-	-
Open heart surgery, n (%)	7 (3.8)	2 (1.9)	0.354
NYHA-FC, n (%)			
I/II	177 (96.7)	107 (100.0)	0.058
III/IV	6 (3.3)	0	0.058
LAV, mL	68.2 ± 25.8	49.7 ± 18.1	<0.001 *
LVEF, %	62.3 ± 7.0	64.0 ± 5.3	0.023 *

BMI = body mass index; LAV = left atrial volume; LVEF = left ventricular ejection fraction; NYHA-FC = New York Heart Association functional class. * Variables with *p* value < 0.05.

**Table 2:** Assessment of angiography images.

	AF (n = 183)	Non-AF (n = 107)	*p* Value
Undetectable VOM on venography in all views, n (%)	15 (8.2)	17 (15.9)	0.044 *
Detectable PLSVA, n (%)	2 (1.1)	2 (1.9)	0.584
Number of branches at VOM ostium			
Type I	143 (78.1)	79 (73.8)	0.477
Type II	25 (13.7)	11 (10.3)	0.477
RAO			
Undetectable VOM on venography in RAO	20 (10.9)	23 (21.5)	0.015 *
VOM-CS angle, °	45.3 ± 20.5	41.4 ± 18.5	0.237
VOM ostium diameter, mm	1.9 ± 0.9	1.7 ± 0.7	0.015 *
CSo diameter, mm	12.8 ± 4.1	11.4 ± 3.7	0.016 *
CS diameter at VOM level, mm	9.1 ± 3.1	8.2 ± 2.9	0.028 *
LAO			
Undetectable VOM on venography in LAO	53 (29.0)	45 (42.1)	0.023 *
VOM-CS angle, °	144.2 ± 36.8	156.0 ± 12.6	0.036 *
VOM-to-CSo distance, mm	37.8 ± 15.6	36.8 ± 13.1	0.684
VOM ostium diameter, mm	1.7 ± 1.0	1.7 ± 0.7	0.664
CSo diameter, mm	11.7 ± 5.8	10.1 ± 5.4	0.022 *
CS diameter at VOM level, mm	7.2 ± 2.9	6.5 ± 2.0	0.047 *
LAO cranial			
Undetectable VOM on venography in LAO + Cranial	39 (21.3)	27 (25.2)	0.442
VOM-CS angle, °	127.9 ± 57.5	109.2 ± 70.7	0.087
VOM-to-CSo distance, mm	39.6 ± 16.6	37.8 ± 13.8	0.419
VOM ostium diameter, mm	1.9 ± 1.1	1.6 ± 0.8	0.069
CSo diameter, mm	13.1 ± 5.0	12.4 ± 4.8	0.263
CS diameter at VOM level, mm	8.1 ± 3.2	7.4 ± 2.4	0.141

AF = atrial fibrillation; CS = coronary sinus; CSo = coronary sinus ostium; LAO = left anterior oblique; PLSVA = persistent left superior vena cava; RAO = right anterior oblique; VOM = vein of Marshall. Other abbreviations as Table 1. * Variables with *p* value < 0.05.

## Data Availability

The datasets presented in this article are not readily available because research data is confidential. Data sharing requests are required to meet the policies of the hospital and the funder. Requests to access the datasets should be directed to doctortangmin@yeah.net.

## Supplementary Materials

The following are available online at https://www.mdpi.com/article/10.3390/jcm11185384/s1, Table S1: Assessment of angiography images according to gender, Table S2: Clinical characteristics after propensity score matching, Table S3: Assessment of angiography images after propensity score matching.
