# Supplementary material for: Angiographic Characteristics of the Vein of Marshall in Patients with and without Atrial Fibrillation
Source: J Clin Med. 2022 Sep 14;11(18):5384. doi: 10.3390/jcm11185384 (PMC9502660; doi:10.3390/jcm11185384)
Supplement: Supplementary file 1 [file jcm-11-05384-s001.zip › jcm-1890277-supplementary.pdf]

Table S1. Assessment of angiography images according to gender

	Female (n = 114)	Male (n = 176)	*P* value
Undetectable VOM on venography in all views, n (%)	14 (12.3)	18 (10.2)	0.586
Detectable PLSVA, n (%)	1 (0.9)	3 (1.7)	0.555
Number of branches at VOM ostium			
Type I	88 (77.2)	133 (75.6)	0.393
Type II	12 (0.5)	25 (14.2)	0.393
RAO			
Undetectable VOM on venography in RAO	17 (14.9)	26 (14.8)	0.974
VOM-CS angle, °	44.8 ± 21.2	43.5 ± 19.1	0.736
VOM ostium diameter, mm	1.7 ± 0.7	1.8 ± 1.0	0.742
CSo diameter, mm	11.6 ± 4.0	12.7 ± 4.0	0.025*
CS diameter at VOM level, mm	8.3 ± 3.0	9.2 ± 3.1	0.016*
LAO			
Undetectable VOM on venography in LAO	39 (34.2)	59 (33.5)	0.904
VOM-CS angle, °	153.0 ± 22.1	144.5 ± 36.4	0.090
VOM-to-CSo distance, mm	37.5 ± 13.4	37.5 ± 15.7	0.987
VOM ostium diameter, mm	1.8 ± 1.0	1.6 ± 0.8	0.122
CSo diameter, mm	10.5 ± 5.1	11.4 ± 6.0	0.124
CS diameter at VOM level, mm	6.7 ± 2.2	7.2 ± 2.9	0.121
LAO cranial			
Undetectable VOM on venography in LAO + Cranial	23 (20.2)	43 (24.4)	0.398
VOM-CS angle, °	122.8 ± 63.4	119.2 ± 63.8	0.471

VOM-to-CSo distance, mm	38.4 ± 14.8	39.3 ± 16.3	0.389
VOM ostium diameter, mm	1.8 ± 1.0	1.8 ± 1.0	0.757
CSo diameter, mm	12.0 ± 4.2	13.4 ± 5.4	0.041*
CS diameter at VOM level, mm	7.4 ± 2.6	8.1 ± 3.1	0.075

CS = coronary sinus; CSo = coronary sinus ostium; LAO = left anterior oblique; PLSVA = persistent left superior vena cava; RAO = right anterior oblique; VOM = vein of Marshall. Other abbreviations as **Table 1**.

*Variables with P value < 0.05.

Table S2. Clinical characteristics after propensity score matching

	AF (n = 76)	Non-AF (n = 76)	*P* value
Age onset, y	55.8 ± 11.7	55.9 ± 12.3	0.857
Age at admission, y	52.3 ± 12.3	50.8 ± 14.7	0.790
Female, n (%)	44 (57.9)	44 (57.9)	0.999
BMI, kg/m^2	26.1 ± 3.6	25.0 ± 3.5	0.073
Comorbidities, n (%)			
Hypertension	38 (50.0)	32 (42.1)	0.329
Diabetes mellitus	10 (13.2)	17 (22.4)	0.137
Coronary heart disease	9 (11.8)	13 (17.1)	0.356
Heart failure	9 (11.8)	4 (5.3)	0.147
Stroke	12 (15.8)	7 (9.2)	0.220
Vascular disease	6 (7.9)	5 (6.6)	0.754
CHA_2DS_2-VASc score	1.9 ± 1.5	-	-
HAS-BLED score	0.5 ± 0.7	-	-
Open heart surgery, n (%)	1 (1.3)	1 (1.3)	0.999
NYHA-FC, n (%)			
I/II	73 (96.1)	76 (100)	0.311
III/IV	3 (3.9)	0	0.311
LAV, ml	68.7 ± 25.3	52.2 ± 19.5	<0.001*
LVEF, %	61.7 ± 6.8	63.3 ± 5.6	0.084

BSA = body surface area; BMI = body mass index; LAV = left atrial volume; LVEF = left ventricular ejection fraction; NYHA-FC = New York Heart Association functional class; PAF = paroxysmal atrial fibrillation;

PerAF = persistent atrial fibrillation. *Variables with P value < 0.05.

Table S3. Assessment of angiography images after propensity score matching

	**AF** (n = 76)	**Non-AF** (n = 76)	***P* value**
Undetectable VOM on venography in all views, n (%)	7 (9.2)	16 (21.1)	0.042*
Detectable PLSVA, n (%)	2 (2.6)	2 (2.6)	0.999
Number of branches at VOM ostium			
Type I	58 (76.3)	54 (71.1)	0.622
Type II	11 (14.5)	8 (10.5)	0.622
RAO			
Undetectable VOM on venography in RAO	9 (11.8)	19 (25.0)	0.036*
VOM-CS angle, °	44.5 ± 19.8	44.6 ± 19.2	0.821
VOM ostium diameter, mm	1.9 ± 0.7	1.7 ± 0.8	0.045*
CSo diameter, mm	12.8 ± 3.8	11.4 ± 3.8	0.040*
CS diameter at VOM level, mm	8.9 ± 2.9	8.4 ± 2.7	0.304
LAO			
Undetectable VOM on venography in LAO	18 (23.7)	32 (42.1)	0.016*
VOM-CS angle, °	143.0 ± 35.5	153.7 ± 12.7	0.092
VOM-to-CSo distance, mm	38.2 ± 14.3	38.0 ± 14.5	0.949
VOM ostium diameter, mm	1.7 ± 1.1	1.7 ± 0.7	0.407
CSo diameter, mm	11.9 ± 5.7	10.3 ± 5.3	0.124
CS diameter at VOM level, mm	7.0 ± 2.3	6.4 ± 1.8	0.211
LAO cranial			

Undetectable VOM on venography in LAO + Cranial	15 (19.7)	23 (30.3)	0.134
VOM-CS angle, °	128.9 ± 55.7	104.6 ± 71.9	0.104
VOM-to-CSo distance, mm	40.5 ± 16.5	38.5 ± 13.9	0.351
VOM ostium diameter, mm	2.0 ± 1.2	1.7 ± 0.8	0.435
CSo diameter, mm	13.4 ± 4.9	12.7 ± 5.2	0.287
CS diameter at VOM level, mm	8.0 ± 2.8	7.2 ± 2.3	0.078

CS = coronary sinus; CSo = coronary sinus ostium; LAO = left anterior oblique; PLSVA = persistent left superior vena cava; RAO = right anterior oblique; VOM = vein of Marshall. Other abbreviations as **Table 1**.

*Variables with P value < 0.05.
